# Practical applicability and effectiveness of passive, back-support exoskeletons in logistics and care: a randomized controlled trial protocol (ELSA LogiCare)

**DOI:** 10.1186/s13063-026-09890-2

**Published:** 2026-07-14

**Authors:** Wendi Sieber, Johannes Voß, Roberto Falz, Max Schuhte, Claudia Wendel, Olaf Ueberschär

**Affiliations:** 1 Department of Rehabilitation Psychology and Clinical Neuropsychology, Magdeburg-Stendal University of Applied Sciences, Stendal, Germany; 2 Department of Human Technology Interaction, Magdeburg-Stendal University of Applied Sciences, Magdeburg, Germany; 3 Institute of Sport Medicine and Prevention, University Leipzig, Leipzig, Germany; 4 Institute for Applied Training Science (IAT), Leipzig, Germany

**Keywords:** Passive exoskeletons, Prevention, Musculoskeletal disorders, Care, Nursing, Logistics, RCT, Open-label

## Abstract

**Background:**

Musculoskeletal disorders (MSDs) are the leading work-related health issue in Europe. While passive back-support exoskeletons have shown promise in reducing physical strain in laboratory settings, their medium-term effects under real-world working conditions remain largely unexplored. This study will assess the applicability and the medium-term effects of passive back-support exoskeletons on MSDs in the logistics and healthcare sectors.

**Methods:**

ELSA LogiCare is a multicenter, parallel-designed, randomized controlled trial. Participants will be randomly assigned in a 2:1 ratio to either an intervention group or a control group. The intervention group will be randomly assigned (1:1) to use one of two passive back-support exoskeleton models for 3 months, while the control group works unassisted as usual. We aim to recruit a total sample size of 120 participants, resulting in approximately 80 participants in the intervention group and 40 participants in the control group. Primary outcomes focus on feasibility and use of the exoskeleton, including daily wearing time. Effectiveness-related outcomes include musculoskeletal complaints after 3 months, assessed using the Cornell Musculoskeletal Discomfort Questionnaire. Secondary outcomes include physical and mental workload (NASA-Raw Task Load Index), job satisfaction (Copenhagen Psychosocial Questionnaire), cognitive performance (Wiener Testsystem), fatigue (Fatigue Scale), acceptance of exoskeletons (Technology Commitment), user satisfaction (Quest 2.0), user experience and implementation barriers (short interviews), absences due to illness, blood pressure, physical activity (Fitbit/Garmin) and dropouts. Outcomes will be assessed at baseline, every 4 weeks during, and immediately after the intervention. The study is open-label, with blinding applied to outcome assessors and data analysts only. Primary analyses will follow the intention-to-treat principle and will be conducted using mixed-effects models with repeated measures to estimate feasibility- and effectiveness-related outcomes over time.

**Discussion:**

When effectively integrated into workplace environments, passive back-support exoskeletons have the potential to significantly alleviate physical strain and enhance efforts to reduce the risks associated with MSDs. Our process evaluation can also help to understand how passive back-support exoskeletons can be successfully integrated into the logistics and care sectors.

**Trial registration:**

German Clinical Trials Register (DRKS) under the DRKS-ID: DRKS00036072.

## Administrative Information

Note: the numbers in curly brackets in this protocol refer to SPIRIT checklist item numbers. The order of the items has been modified to group similar items (see http://www.equator-network.org/reporting-guidelines/spirit-2013-statement-defining-standard-protocol-items-for-clinical-trials/).

**Table:** 

Title {1}	Practical applicability and effectiveness of passive, back-support exoskeletons in logistics and care: a randomized controlled trial protocol (ELSA LogiCare)
Trial registration {2a and 2b}.	The ELSA LogiCare trial has been registered in the German Clinical Trials Register (DRKS) under the DRKS-ID: DRKS00036072. Since October 2008, the DRKS has been recognized as a WHO Primary Registry.
Protocol version {3}	2nd Version. Date of version: February 3, 2026
Funding {4}	This research project is funded within the framework of the "European Regional Development Fund (EFRE)" through resources from the European Union and the state of Saxony-Anhalt, represented by the Investment Bank of Saxony-Anhalt.
Author details {5a}	Wendi Sieber^1†^, Johannes Voß^2,3†^, Roberto Falz^2,3^, Max Schuhte^2^, Claudia Wendel^1^, Olaf Ueberschär^2,4^ ^1^ Department of Rehabilitation Psychology and Clinical Neuropsychology, Magdeburg-Stendal University of Applied Sciences, Stendal, Germany ^2^ Department of Human Technology Interaction, Magdeburg-Stendal University of Applied Sciences, Magdeburg, Germany ^3^ Institute of Sport Medicine and Prevention, University Leipzig, Leipzig, Germany ^4^ Institute for Applied Training Science (IAT), Leipzig, Germany ^†^ - shared first authorship
Name and contact information for the trial sponsor {5b}	European Union Represented by: Investment Bank of Saxony-Anhalt Domplatz 12, 39104 Magdeburg
Role of sponsor {5c}	The funding source had no role in the design of this study and will not have any role during its execution, analyses, interpretation of the data, or decision to submit results.

## Introduction

### Background and rationale {6a}

Musculoskeletal disorders (MSDs) are the most prevalent work-related health issue in Europe [1]. Repetitive movements, unfavorable postures, and frequent or heavy lifting can overload the musculoskeletal system, increasing the risk of developing MSDs [2]. These disorders contribute to high absenteeism rates and result in longer periods of absence compared to other health conditions [1]. The financial costs associated with MSDs in Europe are estimated at approximately 240 billion euros, representing approximately 2% of the EU-15 gross domestic product [3]. Furthermore, MSDs significantly impair the quality of life of affected individuals [4, 5]. In response, the prevention of MSDs has become a priority in European and national occupational safety and health strategies [1].

Given the significant health and economic burden of MSDs, there is a growing demand for effective, scalable workplace interventions. Among emerging ergonomic solutions, passive back-support exoskeletons have gained attention for their potential to mitigate physical strain during manual tasks, especially for lifting tasks [6]. Unlike active exoskeletons, which are heavier and require external power sources, passive exoskeletons are lighter and rely on restoring forces from elastomers or springs for support [7]. Preliminary studies suggest that back-support exoskeletons can be effectively employed in various sectors, including logistics, industry, the military, and healthcare [8–12].

Existing evidence indicates that passive back-support exoskeletons can significantly reduce mechanical load and physical strain, as measured by muscular activity (reduced muscle activity trunk extension SMD: 0.58, *p* = 0.0001; [13]) and metabolic equivalents [12, 14–23]. Subjective parameters, such as self-efficacy, perceived usability, and perceived effort (reduced perceived musculoskeletal strain in back SMD: 0.73, *p* < 0.0001; [13]), also demonstrate positive changes [24–27]. However, the vast majority of studies have been conducted in controlled laboratory settings, using small, homogeneous samples and focusing on immediate physiological effects. There is a critical lack of real-world evidence on medium- and long-term health outcomes, user compliance, and the feasibility and real-world applicability of passive back-support exoskeletons—especially in high-risk sectors such as logistics and healthcare [28, 29]. While laboratory studies suggest beneficial biomechanical effects, little is known about sustained use, user acceptance, and barriers and facilitators to implementation under routine working conditions. A recent meta-analysis highlights the need for methodologically rigorous, prospective longitudinal studies to investigate the health effects of passive back-support exoskeletons [13]. Our study addresses this research gap by employing a prospective, randomized, controlled, and multicentered design, complemented by a pragmatic mixed-methods process evaluation focusing on feasibility, use, and user experience.

### Objectives {7}

This study has two co-primary objectives. The first co-primary objective is to assess the feasibility and practical applicability of passive back-support exoskeletons in the logistics and healthcare sectors under real-world working conditions. Feasibility will be evaluated in terms of intervention adherence (frequency and duration of exoskeleton wearing) and study retention over a 3-month period. In addition, user experience, acceptability, and perceived barriers and facilitators to implementation will be explored through qualitative interviews.

The second co-primary objective is to estimate the effectiveness of passive back-support exoskeleton use on musculoskeletal complaints over a 3-month period compared to usual work without exoskeletons. Effectiveness will be assessed as the change in musculoskeletal complaints measured by the Cornell Musculoskeletal Discomfort Questionnaire (CMDQ).

Secondary objectives include identifying organizational and individual barriers to implementation, evaluating the impact of exoskeleton use on job satisfaction, and exploring potential effects on cognitive performance.

We hypothesize that regular use of passive back-support exoskeletons over 3 months reduces musculoskeletal complaints compared to usual work without exoskeletons.

### Trial design {8}

This is a prospective, multicenter, randomized controlled trial with a waitlist control design and a 1:1:1 allocation ratio to three study arms: control, passive back-support exoskeleton model 1, and passive back-support exoskeleton model 2.

The selection of a 3-month intervention period was based on both physiological and organizational considerations. From a physiological perspective, previous studies on passive back-support exoskeletons have predominantly focused on short-term laboratory-based application or training sequences, often limited to single sessions or a few days of use [12, 14–20]. It has been suggested that potential sustained effects on musculoskeletal outcomes and user adaptation may only become apparent after several weeks of regular use [30]. A 3-month intervention period therefore allows for the assessment of effects beyond immediate or short-term responses while remaining feasible within real-world occupational settings.

From an organizational perspective, the intervention duration was determined by the limited availability of exoskeleton devices and the fixed project timeline. Under these constraints, a 3-month intervention period represented the maximum feasible duration to achieve the planned sample size while ensuring efficient use of available resources. Control participants will continue working as usual during this period and will receive the exoskeleton after completion of the intervention phase; data from this subsequent wearing period will not be included in the statistical analysis.

## Methods: participants, interventions, and outcomes

### Study setting {9}

The trial sites are located in Magdeburg (Magdeburg-Stendal University of Applied Sciences, Department of Engineering and Industrial Design (IWID), Chair of Human-Technology Interaction, Breitscheidstr. 2, 39114 Magdeburg) and Stendal (Magdeburg-Stendal University of Applied Sciences, Department of Applied Human Sciences (AHW), Chair of Clinical Neuropsychology, Osterburger Str. 25, 39576 Stendal). Recruitment and data collection will take place at care providers and logistics companies in Germany. The expected duration of the trial is 3 years, including participant enrollment, intervention implementation, assessments, and data analysis.

### Eligibility criteria {10}

In this trial, employees from the logistics and healthcare sectors working in cooperating institutions will be recruited. For the purpose of this study, logistics occupations are defined as jobs that involve manual handling tasks such as lifting, carrying, or moving goods (e.g., warehouse workers, order pickers, and employees involved in internal transport processes). Healthcare occupations include nursing staff working in inpatient or outpatient settings whose routine duties involve manual patient handling tasks such as lifting, transferring, or repositioning patients.

Prior to study initiation at trial sites, cooperation agreements will be established only with companies in which routine work tasks involve high exposure to manual lifting and carrying loads. High-risk exposure is defined as performing more than 10 lifting or carrying tasks per working day or handling loads exceeding 10 kg per lifting or carrying task.

To ensure that these criteria are met, participating companies will be visited in advance, and relevant departments and job roles will be identified in collaboration with company management and supervisory staff. Only employees working in these predefined departments and roles will be invited to participate in the study.

Eligible participants must have sufficient proficiency in German or English to understand the study information, complete questionnaires, and participate in interviews, as all study instruments are only available and validated in these languages. Exclusion criteria include inability to understand study procedures or to complete required assessments, as well as inability or unwillingness to comply with study procedures over the 3-month intervention period.

The inclusion and exclusion criteria are listed in Table 1. Eligible employees will be recruited and randomized.

**Table 1:** Criteria for inclusion and exclusion

Inclusion criteria	Exclusion criteria
• Workers from the logistics or healthcare sectors whose regular occupational tasks include recurring manual lifting or carrying activities • High-risk exposure to lifting and carrying loads, defined as performing more than 10 lifting or carrying tasks per working day or handling loads exceeding 10 kg per lifting or carrying task • Age over 18 years • Sufficient proficiency in German or English to understand study procedures and complete questionnaires and interviews	• Acute viral/bacterial infections and infectious diseases • Orthopaedic, rheumatological, cardiovascular, or psychiatric conditions that prevent the use of passive exoskeletons • Inability to understand study procedures or to complete questionnaires and required assessments • Inability or unwillingness to comply with study procedures over the 3-month intervention period • Pregnancy • Other conditions that lead to severe physical or psychological limitations

### Who will take informed consent? {26a}

Informed consent will be obtained by trained research staff members. During a personal conversation, the research staff members will provide detailed explanations about the study objectives, purpose, procedures, harms, and participants’ rights. At this stage, employees will receive written information as well as detailed informed consent. After an appropriate period for consideration, participants will have the opportunity to ask questions, verification of inclusion and exclusion criteria will be conducted, and written consent will be obtained.

### Additional consent provisions for collection and use of participant data and biological specimens {26b}

N/A. No biological specimens were collected as part of this trial.

## Interventions

### Explanation for the choice of comparators {6b}

The comparator in this trial is standard care without the use of a back-support exoskeleton. This choice reflects the current standard practice in logistics and nursing professions, where no assistive exoskeletal devices are typically used. By comparing the use of a passive back-support exoskeleton to usual practice, the study aims to evaluate the added value of the intervention under real-world conditions. This comparison allows for a clear assessment of the effectiveness, usability, and safety of the device relative to the current occupational norms.

### Intervention description {11a}

Participants allocated to the intervention condition receive one of two passive back-support exoskeleton models (Model A or Model B) for 3 months, assigned randomly. This is accompanied by regular follow-up appointments and asynchronous educational materials on its use. This includes monitoring daily wearing time and frequency of use per week. Participants are instructed to wear the exoskeleton “as frequently as possible” during their daily work activities over the 3-month period. This instruction was deliberately chosen to obtain a realistic and practice-oriented representation of actual usage behavior in everyday work. Since occupational activities typically encompass a broad range of tasks (e.g., sitting, standing, lifting), participants are instructed to use the exoskeleton during at least 75% of all lifting tasks (see also primary applicability endpoint). No additional strategies to promote or enforce adherence are implemented, as justified non-use in this context provides important insights and represents a relevant aspect of ecological validity.

Daily wearing time will be assessed using a combination of self-reported wearing logs and objective sensor-based data. Participants will complete a daily wearing protocol documenting the duration and frequency of exoskeleton use during their work shifts throughout the 3-month intervention period.

In addition, the exoskeleton is equipped with an integrated motion sensor that records movement patterns indicative of exoskeleton use. Sensor-based measurements will be conducted during predefined measurement periods (selected full working weeks) rather than continuously throughout the entire intervention period. These data will be used to complement and validate self-reported wearing time and to assess the consistency and plausibility of self-reported usage patterns.

The control group will not receive an exoskeleton and will continue with their usual work. Participants in the control group will be instructed to continue their usual work practices and to refrain from using back braces or other back-support devices during the 3-month study period, unless medically indicated. Any medically necessary treatment or therapeutic measure may be initiated or continued at any time and will be documented.

During the intervention period, several assessments will be conducted at 4-week intervals. These assessments will include questionnaires and brief interviews to identify entry barriers, acceptance, and potential barriers to implementing exoskeletons in daily work routines, as well as to assess musculoskeletal complaints, fatigue, job satisfaction, and cognitive performance. A detailed list of the constructs to be assessed and their corresponding measurement methods is provided in Table 2. The study visits for data collection are identical in both groups.

**Table 2:** Outcomes and operationalization

Outcomes	Data collection, operationalization
*Outcome evaluation*	
• Musculoskeletal complaints	• Cornell Musculoskeletal Discomfort Questionnaire, German version (D-CMDQ) [31]
• Sick days • Medical costs (estimate)	• Number of total sick days • Number of musculoskeletal-related sick days • Medical costs per musculoskeletal-related sick day
• Physical and mental workload • Work-related exhaustion • Job satisfaction	• NASA-Raw Task Load Index, German version (NASA-RTLX) [32] • Fatigue Scale (FS) [33] • Copenhagen Psychosocial Questionnaire, German version (COPSOQ) [34] • Resting heart rate (blood pressure monitor, Boso medicus) • Resting blood pressure (blood pressure monitor, Boso medicus)
• Attention and memory performance due to low physical exhaustion (subgroup, nursing)	• Vienna Test System (WTS) [35]
*Process evaluation*	
• Applicability of the exoskeletons in real-world work settings	• Wearing time of the exoskeleton in relation to the total time of daily physical activity
• Utilization of the exoskeletons • Acceptance among participants and employers • User satisfaction • User experience and implementation barriers	• Frequency and duration of wearing per week • Technology readiness short scale (TB) [36] • Quebec User Evaluation of Satisfaction with Assistive Technology 2.0 (QUEST 2.0) [37] • Brief interview

After the intervention, the control group will also be allowed to use the exoskeleton for 3 months (waiting-control design). However, this usage period will not be included in the statistical analysis.

A crossover design was deliberately not chosen due to important methodological and practical limitations in this occupational setting. Although laboratory studies suggest that exoskeleton use can acutely influence movement patterns, muscle activation, and perceived workload, evidence regarding the persistence and reversibility of such changes under real-world working conditions is currently limited. In particular, no evidence-based washout period can be defined. Consequently, potential carryover effects cannot be reliably excluded. To minimize the risk of residual behavioral or motor adaptations compromising internal validity, a parallel-group design was considered the more appropriate and methodologically robust approach to address the research question.

### Criteria for discontinuing or modifying allocated interventions {11b}

No modifications are planned for the allocated intervention. The aim of the study is to assess the practical applicability of the exoskeleton under real-world working conditions. Should participants wish to discontinue the intervention—for example, due to discomfort or other subjective complaints—such discontinuations will be considered important study outcomes. These decisions provide valuable insights into the acceptability and user experience of the exoskeleton.

### Strategies to improve adherence to interventions {11c}

To support compliance and adherence, research staff will be available on request throughout the intervention period to assist participants, answer questions, and verify correct exoskeleton use. Upon enrollment, participants receive standardized information and training on the purpose of the study, correct use of the exoskeleton, and study procedures.

No additional strategies to actively promote or enforce adherence (e.g., reminders, incentives, real-time feedback on wearing time, or communication of usage data to workplaces or supervisors) are implemented. This decision was made deliberately to avoid influencing natural usage behavior and to preserve ecological validity by capturing realistic patterns of exoskeleton use under routine working conditions.

### Relevant concomitant care permitted or prohibited during the trial {11d}

N/A. No specific concomitant care is prohibited during the trial.

### Provisions for post-trial care {30}

As the intervention involves the use of a passive back-support exoskeleton with minimal risk, no specific post-trial care is planned. Participants will be informed about the study results upon request. After completion of the study, all participants will return the exoskeletons to the study team; no post-trial access to the device is provided.

### Outcomes {12}

Primary and secondary outcomes are listed below. To ensure confidentiality of personal information, participants will be assigned numerical study codes. Table 3 summarizes all measurement time points.

**Table 3:** Schedule of enrollment, interventions, and assessments

		**Study period**				
	**Screening**	**Baseline**	**Call**	**1 month**	**2 months**	**Final examination**
**Examination**	**−t**_**1**_	**t**_**0**_		**t**_**1**_	**t**_**2**_	**t**_**3**_
**Time (weeks)**	−2	0	1	4	8	12
**Socio-demographic and clinical data**						
Inclusion and exclusion criteria	**x**					
Written consent		**x**				
Randomization		**x**				
Demographic data and medical history		**x**				
Exoskeleton training		**x**	**x**			
**Evaluation of effects**						
Musculoskeletal complaints (D-CMDQ)		**x**				**x**
Number of sick days (total, musculoskeletal)		**x**		**x**	**x**	**x**
Work armouring (NASA-RTLX)		**x**		**x**	**x**	**x**
Fatigue (FS)		**x**		**x**	**x**	**x**
Work-related psychosocial stress (COPSOQ)		**x**				
Job satisfaction (Item B11.7 COPSOQ)		**x**		**x**	**x**	**x**
Cognitive performance (Vienna Test System)^a^		**x**				
Resting heart rate^b^		**x**				**x**
Resting blood pressure^b^		**x**				**x**
**Evaluation of applicability**						
Wearing frequency (per week)				**x**	**x**	**x**
Acceptance (TB)		**x**				
Usability (QUEST 2.0)				**x**	**x**	**x**
Implementation barriers (free text, optional)		**x**		**x**	**x**	**x**
Barriers to implementation (interview)				**x**		**x**
**Intervention group (IG)/control group (CG)**						
Use of exoskeletons (IG)						
Normal workflow (CG)						

*Abbreviations*: *D-CMDQ* Cornell Musculoskeletal Discomfort Questionnaire (German), *NASA-RTLX* NASA-Raw Task Load Index, *FS* Fatigue Scale, *COPSOQ* Copenhagen Psychosocial Questionnaire, *TB *Brief Technology Readiness Scale, *QUEST 2.0* Quebec User Evaluation of Satisfaction with Assistive Technology 2.0, *IG* intervention group, *CG* control group

^a^Cognitive performance: pre-post-measurement during one shift *with* and one shift *without* exoskeleton

^b^heart rate and blood pressure: at least 2 measurements per day (morning, evening) for 3 days

#### Co-primary endpoints

Two co-primary endpoints are defined: (1) an intervention adherence endpoint reflecting feasibility under real-world working conditions, and (2) an effectiveness-related endpoint assessing changes in musculoskeletal complaints. As this is a feasibility-oriented trial, effectiveness outcomes will be analyzed using an estimation-focused approach to quantify effect sizes and variability rather than to provide confirmatory hypothesis testing.

##### Co-primary intervention adherence endpoint

Intervention adherence will be evaluated based on predefined compliance and retention criteria. Task-level compliance will be quantified as the proportion of lifting-task time during which the exoskeleton is worn, with a target threshold of ≥ 50%. In addition, overall wearing time in relation to daily physical activity will be assessed using an objective proxy measure derived from smartwatch-based movement recordings attached to the exoskeleton (Garmin Vivoactive 4).

Study-level adherence (retention) will be assessed as the proportion of participants who remain in the study and provide outcome data through the final 3-month assessment, with a target retention rate of ≥ 75% (i.e., dropout ≤ 25%). While dropout of up to 50% is conservatively anticipated for planning purposes, retention ≥ 75% represents the desired feasibility target.

##### Co-primary effectiveness-related endpoint

The co-primary effectiveness-related endpoint is the change in musculoskeletal complaints from baseline to 3 months, assessed using the Cornell Musculoskeletal Discomfort Questionnaire (CMDQ) score, comparing the intervention group with the control group.

This endpoint will be analyzed using longitudinal models to estimate mean changes, effect sizes, and 95% confidence intervals. Given the feasibility focus of the trial, these analyses are intended to generate preliminary evidence and inform the design and sample size calculation of future adequately powered confirmatory studies. No formal multiplicity adjustment will be applied, as the trial is not powered for confirmatory testing.

##### Cornell Musculoskeletal Discomfort Questionnaire, German version (D-CMDQ) [31]

The “Cornell Musculoskeletal Discomfort Questionnaire” was developed in 1999 by Hedge et al. [38] and, in 2016, adapted by Kreuzfeld et al. [31] as a German-language version and validated (D-CMDQ). The questionnaire measures the subjectively perceived frequency and intensity of musculoskeletal pain and complaints, along with work-related impairments, for 20 body regions. It is economical, applicable to many occupations, and can be tested as individual or group.

Reliability: Internal consistency was assessed using Cronbach’s alpha for the frequency, severity, and work disorder subscales. This resulted in acceptable internal consistencies of α_frequency_ = 0.75, α_severity_ = 0.77 and α_work disorder_ = 0.82 [31].

Validity: To test convergent validity, the correlation between the D-CMDQ and the Numeric Rating Scale (NRS) was determined. According to Spearman, this resulted in medium to highly positive correlation coefficients [31].

Minimally clinically important difference/minimal detectable change: no information available.

#### Secondary outcome measurements and endpoints

##### National Aeronautics and Space Administration - Raw Task Load Index, German version (NASA-RTLX) [32]

The German version of the “NASA-RTLX” is based on the English-language long version by Hart & Staveland [39]. The questionnaire measures subjective workload on the subscales of mental, physical, and time demands as well as subjectively perceived performance, effort, and frustration. In the shortened version of the Raw-TLX questionnaire, the second part, which involves individual weighting of the subscales, is omitted.

Reliability: A Cronbach’s alpha of *α* = 0.84 indicates that the overall workload scale is highly reliable [40].

Validity: Positive results were observed regarding convergent, discriminant, and factorial validity [40].

Minimally clinically important difference/minimal detectable change: 15.8 to 24.3 points [41]

##### Fatigue Scale (FS) [33]

The “Fatigue Scale” is a German adaptation of the English version developed by Trudie Chalder and Simon Wessely [42]. It records fatigue levels and differentiates among three subscales based on the severity of physical and mental fatigue symptoms.

Reliability: High internal consistency was assessed for all scales: Cronbach´s alpha: α_total severity_ = 0.93, α_physical fatigue_ = 0.92, and α_mental fatigue_ = 0.86 [33].

Validity: Positive results were observed regarding convergent, discriminant, and factorial validity [33].

Minimally clinically important difference/minimal detectable change: no information available.

##### Copenhagen Psychosocial Questionnaire, German version (COPSOQ) [34]

The “Copenhagen Psychosocial Questionnaire” was originally developed by a Danish research team [43] and adapted by the Freiburg Research Centre for Occupational Sciences GmbH (FFAW) as a German-language version [34]. It was developed to measure psychosocial stress in an occupational context across all occupations and sectors. It also functions as a screening procedure for assessing psychological hazards in the workplace and, therefore, fulfills the test statistical quality criteria required by DIN EN ISO 10075-3. The German version is “available to all companies free of charge.” [34].

Reliability: Cronbach’s alpha for each subscale indicated good reliability (*α* = 0.68 to *α* = 0.89) [34].

Validity: Positive results were observed regarding convergent, discriminant, and factorial validity [34].

Minimally clinical important difference/minimal detectable change: 0.8 to 5.9 points [44]

##### Vienna Test System for cognitive performance diagnostics (WTS) [35]

The Vienna Test System (WTS) is an advanced software solution designed for psychological performance diagnostics. It is a widely used instrument that has proven to be reliable and valid in various studies. The WTS is used in areas such as neuropsychological diagnostics, aptitude diagnostics, and research [35].

Three tests for cognitive performance diagnostics are used in the ELSA LogiCare trial. The *SPAN method* measures verbal working memory and enables differentiated categorization of test subjects across a broad performance spectrum [45]. The *TACO* assesses selective attention, long-term concentration maintenance, and divided attention skills. [46]. The *Trail Making Test* (TMT) measures the speed of visuomotor processing and cognitive flexibility [47].

Minimally clinically important difference/minimal detectable change: no information available.

##### Short Scale Technology Commitment (TB) [36]

The “Short Scale Technology Commitment” aims to predict the effective use of new technologies. It subjectively measures three key determinants: technology acceptance, technology competence, and technology control beliefs. The scale, which consists of 12 items, is cost-effective and not restricted to any particular technology.

Reliability: With a Cronbach’s alpha of α = 0.84 in each case, the overall scale, the technology acceptance subscale, and the technology competence subscale demonstrated high reliability. An acceptable internal consistency of *α* = 0.74 was determined for the technology control subscale [36].

Validity: Three validation studies observed positive results regarding convergent, discriminant, and incremental validity [36].

Minimally clinically important difference/minimal detectable change: no information available.

Quebec User Evaluation of Satisfaction with Assistive Technology 2.0 (QUEST 2.0) [37]

The German version of the “Quebec User Evaluation of Satisfaction with Assistive Technology 2.0” is based on the English version of the same name by Demers et al. [37]. The questionnaire assesses satisfaction with an assistive device through two subscales: satisfaction with the device itself and satisfaction with the related service.

Reliability: For internal consistency, acceptable values of Cronbach’s alpha *α* > 0.7 were determined [48].

Validity: Positive results were observed regarding convergent, discriminant, and factorial validity [37].

Minimally clinically important difference/minimal detectable change: no information available.

### Participant timeline {13}

Trained research staff members will provide detailed explanations about the study objectives, processes, harms, and participants’ rights. They will respond to any questions from participants or representatives and ensure a complete understanding of the information provided. Once participants have successfully enrolled in the study, they will be randomly assigned to either the control or intervention group, followed by a baseline assessment.

**Fig. 1:**
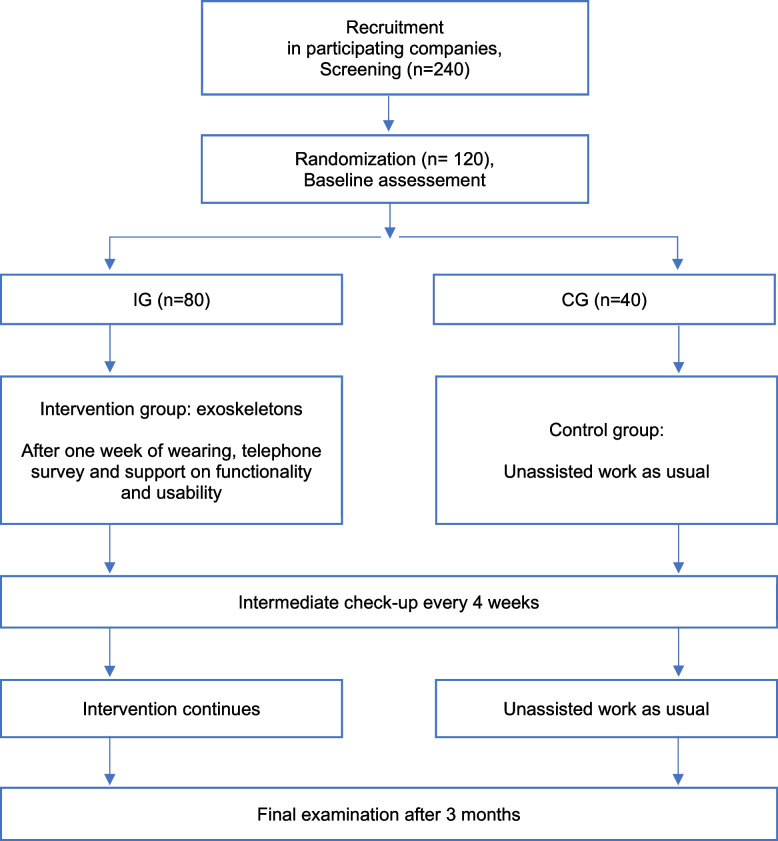
Flow chart. The trial is conducted using a waiting-control design. The control group receives the exoskeletons after the intervention period for 3 months. The wearing time of the control group is not shown in the flow chart as it is not included in the analysis

The study visits for data collection are identical in both groups. During the intervention period, four assessments will be conducted: a baseline assessment with medical history collection, instructions, and training on the correct use of the exoskeleton; two follow-up assessments with questionnaires and short interviews administered at 4-week intervals; and a final assessment conducted 3 months later (Fig. 1).

### Sample size {14}

This study is designed as a feasibility-oriented randomized controlled trial with a primary focus on intervention adherence under real-world working conditions. Primary feasibility outcomes include the following: (1) *task-level compliance with exoskeleton use* (worn for at least 50% of the time during lifting tasks) and (2)* study-level adherence* (defined as at least 75% retention through the 3-month assessment). The sample size is therefore primarily determined by feasibility considerations, including the availability of exoskeleton devices, recruitment capacity within cooperating companies, and the fixed project timeline.

Due to the lack of comparable prospective long-term studies on passive back-support exoskeletons in occupational settings, a formal a priori sample size calculation based on expected effect sizes for musculoskeletal outcomes was not feasible. Consequently, the study is not powered to provide confirmatory evidence regarding effectiveness-related outcomes.

Participants will be randomized using a 2:1 allocation ratio (intervention to control). The recruitment target is defined in terms of evaluable participants: 80 complete datasets in the intervention group and 40 complete datasets in the control group (total *n* = 120 complete datasets). A complete dataset is defined as outcome data available at baseline, at two intermediate follow-up assessments after 1 and 2 months, and at the final assessment after 3 months.

Given the real-world occupational setting and the multi-visit follow-up schedule, substantial attrition is anticipated. Assuming a conservative dropout rate of up to 50%, achieving 120 complete datasets may require randomizing up to approximately *N* = 240 participants (≈160 intervention, ≈80 control). Recruitment will therefore continue until these complete-dataset targets are reached, or until a maximum of *N* = 240 participants have been randomized, whichever occurs first, subject to feasibility constraints (e.g., device availability, company participation, and project timeline). The recruitment stopping rule is based on follow-up completeness and feasibility considerations and is not linked to interim effectiveness results.

Effectiveness-related outcomes will be analyzed using longitudinal models to estimate effect sizes, variability, and 95% confidence intervals rather than to formally test hypotheses. These estimates, together with feasibility parameters, will be used to inform the design and sample size calculation of future adequately powered confirmatory trials.

### Recruitment {15}

We assume that cooperation agreements will have to be concluded with several companies and hospitals to achieve the planned number of cases. It is planned to conduct three survey waves, each lasting 6 months (3 months of intervention followed by 3 months of exchange in the waiting-control design). As a result, a recruitment period totalling 18 months is planned.

Before the cooperation agreements are finalized, site visits will be conducted at the companies and hospitals. During the first contact between employees of the participating companies and the trial staff, information will be provided regarding the purpose and procedures of the trial. At this stage, employees will receive written information as well as detailed informed consent. After an appropriate period for consideration, participants will have the opportunity to ask questions, verification of inclusion and exclusion criteria will be conducted, and written consent will be obtained. Following this, participants will be enrolled in the trial, and the scheduling of the baseline assessment will occur.

## Assignment of interventions: allocation

### Sequence generation {16a}

Using a randomization software (https://www.randomizer.at), participants will be randomized in a 1:1:1 allocation ratio to three study arms: control, passive back-support exoskeleton model 1, and passive back-support exoskeleton model 2. Randomization will be stratified by sector (logistics or healthcare), trial site (participating cooperation partners), and sex (women or men) to ensure balanced group allocation across occupational contexts. Permuted block randomization with a block size of 6 will be used.

### Concealment mechanism {16b}

Allocation concealment will be ensured through centralized computer-based randomization. Group assignment is generated by the randomization software only after a participant has been enrolled and baseline eligibility has been confirmed. Investigators involved in recruitment and enrollment will not have access to the upcoming allocation sequence.

Due to the visible and physical nature of the intervention (use of a back-support exoskeleton), blinding of participants and intervention personnel is not feasible.

### Implementation {16c}

The random allocation sequence is generated by a member of the study team who is not involved in participant enrollment or data collection.

Participants are enrolled by trained research staff at the respective study sites.

Group assignment is conducted automatically by the randomization software after enrollment, ensuring allocation concealment.

## Assignment of interventions: blinding

### Who will be blinded {17a}

This study is designed as an open-label randomized controlled trial, as blinding of participants and staff delivering the intervention is not feasible due to the visible and physical nature of the exoskeleton. Consequently, neither participants nor those overseeing the intervention will be blinded. However, to mitigate potential bias, outcome assessors and data analysts will remain blinded to group assignment, and all data will be pseudonymized prior to analysis to ensure objectivity. We acknowledge the risk of performance bias and detection bias, particularly in subjective outcome measures such as perceived workload, fatigue, or job satisfaction. To address these risks, we use validated and standardized questionnaires to reduce the influence of personal interpretation. Furthermore, participants in both arms will receive comparable levels of researcher attention and contact, helping to minimize differential expectations or behavior changes that could otherwise influence the results.

### Procedure for unblinding if needed {17b}

N/A. Since only data analysts and outcome assessors are blinded, unblinding during the trial is generally not required.

## Data collection and management

### Plans for assessment and collection of outcomes {18a}

No formal pre-screening data are collected prior to enrollment. Recruitment is based on voluntary participation within cooperating companies. Interested employees receive detailed study information and are enrolled after providing written informed consent, provided that inclusion criteria are met.

Baseline data collection is initiated after enrollment. Outcome assessments will be conducted on-site at the participating companies and hospitals. Data collection includes sociodemographic characteristics and a brief personal medical history obtained through an open-ended questionnaire administered at baseline.

Outcome parameters are documented in standardized data sheets and entered into a secure database. Table 3 provides a detailed schedule of participation and measurement time points, including the constructs to be assessed and the corresponding validated instruments. All assessments will be conducted and monitored by trained study personnel at predefined time points to ensure the quality, consistency, and comparability of the collected data.

### Plans to promote participant retention and complete follow-up {18b}

No changes are planned for the assigned intervention, as the study aims to evaluate the practical usability of the exoskeleton under real-world working conditions. If participants discontinue the intervention (e.g., due to discomfort), these decisions will be considered meaningful outcomes that provide valuable insights into user acceptance.

To encourage participant engagement and promote data completeness, questionnaires are administered using the online survey platform LimeSurvey [49]. For each assessment, participants receive an email invitation containing a personalized survey link. Participants have a 1-week period to complete each questionnaire. If a questionnaire has not been completed within 5 days, an automated reminder email is sent. Within the questionnaire, most items are mandatory and participants cannot proceed without providing a response, thereby minimizing item-level missing data.

Any study dropouts and instances of non-response at the questionnaire level will be documented. All collected data will be included in the analyses in accordance with the statistical methods described below.

### Data management {19}

The trial management team develops the case report forms (CRFs) and provides them to the participating centers in electronic format (eCRFs) using the LimeSurvey application [49].

Source Data, according to ICH-GCP E6, includes all information from original records and copies of original records of findings, observations, or other activities within the trial that are required for traceability and recording of variables. The original data is contained in the original documents (original records or certified copies), and its origin can be traced through the case file (electronically or in paper form). The data collected will be reviewed by two researchers and organized for statistical evaluation.

### Confidentiality {27}

Information about participants will be collected, communicated, and stored with the highest level of confidentiality during the trial. Each participant will be assigned a unique identification number to ensure their anonymity in all related documents and data. Information will be stored in secure electronic databases protected by passwords, while physical documents will be kept in locked cabinets accessible only to authorized research staff and monitors. All data are collected, stored, and processed by the European data protection regulation.

The data is stored on Magdeburg-Stendal University of Applied Sciences servers. At the end of the trial, the database will be locked (database lock). After that, any changes to the data in the database can only be made with a written agreement signed by both the trial management and the data manager. Personal information about participants will not be revealed in any publications or reports.

### Plans for collection, laboratory evaluation and storage of biological specimens for genetic or molecular analysis in this trial/future use {33}

N/A. No biological specimens were collected as part of this trial.

## Statistical methods

### Statistical methods for primary and secondary outcomes {20a}

Data will be analyzed using IBM SPSS Statistics (Version 29; IBM, Armonk, New York, USA). For the primary effectiveness analysis, both exoskeleton arms (Exo1 and Exo2) will be pooled into a single intervention group and compared to the control group. Exploratory analyses will additionally compare the three randomized arms separately. The primary analysis will follow an intention-to-treat (ITT) approach, meaning that all randomized participants will be analyzed in the groups to which they were initially assigned. Data from participants with missing information will be analyzed under the "missing at random" assumption. Per-protocol analyses will include only those participants in the intervention group who wore the exoskeleton at least 75% of the days each week when lifting tasks are performed. These analyses will be conducted as sensitivity analyses to assess the robustness of effectiveness-related estimates with respect to intervention adherence and wearing behavior. 

The primary applicability endpoint will be assessed descriptively. To evaluate the metric endpoints, mixed-effects models with a repeated measures structure will be estimated using restricted maximum likelihood. In this model, the measured values collected during all study visits will be considered the dependent variable. We will include treatment group (exoskeleton vs. control) and categorical time as fixed effects. Interactions between the group and time (categorical) will also be modeled. For random effects, we will include an intercept for each subject. Within the mixed models, we will estimate 95% confidence intervals (CIs) and *p*-values for contrasts between groups for the 3-month period. For sensitivity analyses, only patients with complete paired baseline and 3-month follow-up data will be included, ensuring a time difference within groups is analyzed using a paired *t*-test for dependent samples.

### Interim analyses {21b}

No interim analysis is planned.

### Methods for additional analyses (e.g., subgroup analyses) {20b}

In exploratory analyses, the original three randomization arms will be entered as fixed effects. Additional exploratory subgroup analyses may be conducted to investigate differences in outcomes between sectors (logistics vs. healthcare), or by participant characteristics such as age or sex. These analyses will be reported as exploratory.

### Methods in analysis to handle protocol non-adherence and any statistical methods to handle missing data {20c}

Protocol non-adherence (e.g., early discontinuation of the intervention) will be documented and analyzed descriptively. Data from participants with missing values will be included under the assumption that the missing values are missing at random.

### Plans to give access to the full protocol, participant-level data, and statistical code {31c}

Upon reasonable request, access to the full study protocol, anonymized participant-level data, and statistical analysis code will be made available after publication of the primary study results. Requests will be reviewed by the study steering committee to ensure compliance with ethical and legal data protection requirements.

## Oversight and monitoring

### Composition of the coordinating center and trial steering committee {5d}

No independent trial steering committee has been established for this low-risk trial. Oversight, academic leadership, and data management responsibilities are handled by the coordinating team at Magdeburg-Stendal University of Applied Sciences, as detailed below:

Academic management and data management:

Prof. Dr. rer. nat. Olaf Ueberschär

Chair of Human-Technology Interaction, Magdeburg-Stendal University of Applied Sciences

Breitscheidstraße 2, 39114 Magdeburg, Deutschland

E-mail: olaf.ueberschaer@h2.de

Prof. Dr. Claudia Wendel

Chair of Clinical Neuropsychology, Magdeburg-Stendal University of Applied Sciences.

Osterburger Straße 25, 39576 Stendal, Deutschland

E-mail: claudia.wendel@h2.de

Operational management:

M. Sc. Johannes Voß

Chair of Human-Technology Interaction, Magdeburg-Stendal University of Applied Sciences

Breitscheidstraße 2, 39114 Magdeburg, Deutschland

E-mail: johannes.voss@h2.de

### Composition of the data monitoring committee, its role and reporting structure {21a}

No independent data monitoring committee (DMC) is established for this low-risk study. All procedures, including the development of the study protocol, the case report form, the content of participant information and consent, the application for ethics approval, data processing, central and on-site monitoring, and analyses, follow standard operating procedures (SOPs). The coordinating center is the Chair of Human-Technology Interaction, Magdeburg-Stendal University of Applied Sciences, and is responsible for data monitoring. Safety and data adequacy will be monitored every 2 weeks under the supervision of the principal investigator. During monitoring visits, the monitor will:

(1) Verify the informed consent forms; (2) Conduct a source data review of key data (e.g., primary and secondary endpoints, adverse events); (3) Discuss any open questions that have arisen during data management. Additional regular teleconferences and meetings will be held as needed to ensure compliance with appropriate quality standards and timelines.

### Adverse event reporting and harms {22}

The interventions in this trial are expected to carry low risk. Potential adverse events related to the use of the passive back-support exoskeleton may include transient discomfort, pressure, friction, or skin irritation at points of force transmission (e.g., shoulders or hips), as well as musculoskeletal soreness associated with adaptation to wearing the device. Improper adjustment of the exoskeleton to individual body proportions may increase the likelihood of such events.

To minimize these risks, a lightweight and ergonomically adjustable exoskeleton model will be used that does not restrict the user’s range of motion. Participants will receive comprehensive initial training on correct adjustment and use of the exoskeleton, with regular checks by trial staff to ensure proper fit and safe use.

Adverse events (AEs) are defined as any unfavorable or unintended signs, symptoms, or injuries occurring during the study period, regardless of whether they are considered related to the intervention. This includes events potentially related to changes in work behavior during trial participation (e.g., musculoskeletal complaints, minor injuries, or falls occurring during routine work activities). Serious adverse events (SAEs) include events that result in death, life-threatening conditions, hospitalization, persistent or significant disability, or are otherwise considered medically serious.

All AEs and SAEs, whether solicited or spontaneously reported, as well as any unintended effects occurring from enrollment until the end of the trial, will be documented. Documentation will include onset, duration, severity, management, outcome, and the investigator’s assessment of relationship to exoskeleton use. Serious adverse events will be reported to the responsible ethics committee within 24 h of the research team becoming aware of the event.

### Frequency and plans for auditing trial conduct {23}

N/A. No independent auditing of trial conduct is planned, as the study is considered low risk.

### Plans for communicating important protocol amendments to relevant parties (e.g., trial participants, ethical committees) {25}

Any important protocol amendments (e.g., changes to eligibility criteria, outcomes, or procedures) will be submitted for review and approval by the responsible ethics committees prior to implementation. If applicable, updated information will be provided to trial participants. All amendments will be documented in the trial registry and communicated to the study team via formal notification channels.

### Dissemination plans {31a}

A summary of the results will be individually presented to each participating company or hospital. Each participant receives an individual results report upon request. In addition, the findings will be published in peer-reviewed journals and presented at both national and international conferences and seminars.

The authorship criteria are based on the guidelines of the International Committee of Medical Journal Editors (ICMJE), which state that authors must meet the following requirements:

- Substantial contributions to the conception, design, data collection, analysis, or interpretation of the study.
- Drafting or revising the manuscript critically for important intellectual content.
- Final approval of the version to be published and agreement to be accountable for all aspects of the work.

No professional writers or medical writers will be used in the preparation of this manuscript. All authors have independently contributed to the manuscript and have approved the final version for submission.

## Discussion

This trial aims to evaluate both the effectiveness of passive back-support exoskeletons and selected aspects of their practical use under real working conditions. While the primary effectiveness outcome focuses on musculoskeletal health, aspects related to practical use such as adherence, user acceptance, and perceived implementation barriers are assessed using quantitative adherence measures and qualitative user feedback.

However, the study does not include a formal, theory-driven process evaluation. This represents a major limitation and restricts the extent to which implementation processes, contextual factors, and organizational influences can be comprehensively understood. As a result, conclusions regarding implementation and real-world integration of exoskeletons must be interpreted cautiously. Although adherence data and qualitative interviews provide exploratory insights into user experience and feasibility, they do not fully capture the complexity of implementation processes across different workplace settings.

Several studies have demonstrated that passive back-support exoskeletons reduce physical strain under laboratory conditions [16–18, 50, 51]. However, it remains unclear whether these acute physiological effects translate into sustained health-related outcomes, such as reduced musculoskeletal complaints, in occupational settings. The ELSA LogiCare trial addresses this gap by providing medium-term data collected under real-world working conditions, while explicitly acknowledging its limitations with regard to implementation analysis.

It can be assumed that exoskeletons can only have a preventive effect if worn continuously. However, barriers to successful implementation remain high [52]. Previous implementation studies have indicated that perceived discomfort, workflow interference, and a lack of perceived benefit are major predictors of low adherence [53, 54]. By examining wearing behavior and collecting qualitative user feedback, this study provides preliminary insights into these factors. However, future studies incorporating comprehensive process evaluation frameworks will be necessary to systematically examine these mechanisms.

Sector-specific challenges further influence feasibility and implementation. In the logistics sector, difficulties in recruiting participants for a 3-month intervention period may arise due to high employee turnover, particularly in large companies employing temporary or seasonal workers [55]. In contrast, the healthcare sector presents different challenges related to the highly variable nature of daily work tasks. Physically demanding activities such as patient lifting during mobilization or emergency situations alternate with less physically demanding tasks, including documentation or patient transfers. During these latter activities, the exoskeleton may be perceived as disruptive, raising questions about repeated donning and doffing throughout the workday. In addition, hygiene requirements represent a critical consideration in healthcare settings and must be carefully addressed prior to implementation.

Despite these limitations, to our knowledge, ELSA LogiCare is among the first randomized controlled trials to investigate the medium-term effects of passive back-support exoskeletons under real-world working conditions. If shown to be effective and feasible, exoskeletons could complement existing ergonomic interventions and contribute to the development of evidence-based recommendations and occupational health policies. Furthermore, the findings may inform manufacturers, employers, and policymakers and support the design of future trials that integrate effectiveness evaluation with comprehensive process evaluation approaches.

## Trial status

This protocol represents the second version of the study protocol, finalized on February 3, 2026. Recruitment commenced in January 2026, and the first participants have been successfully enrolled. Recruitment is ongoing and is expected to be completed by June 2027.

## Data Availability

The datasets that will be generated and/or analyzed during the trial will be available from the corresponding author upon reasonable request.

## Abbreviations

CG: Control group
COPSOQ: Copenhagen Psychosocial Questionnaire
D-CMDQ: Cornell Musculoskeletal Discomfort Questionnaire
eCRF: Electronic case report forms
EFRE: European Regional Development Fund
ELSA LogiCare: Exoskeletons in Saxony-Anhalt in Logistics and Care
EMG: Electromyography
EU-15: European Union
FS: Fatigue Scale
IG: Intervention group
MSDs: Musculoskeletal Disorders
NASA-RTLX: NASA-Raw Task Load Index, German version
QUEST 2.0: Quebec User Evaluation of Satisfaction with Assistive Technology 2.0
SOPs: Standard operating procedures
TB: Technology readiness short scale, German version
WTS: Vienna Test System
